# Toxicity and genotoxicity of wastewater from gasoline stations

**DOI:** 10.1590/S1415-47572009005000094

**Published:** 2009-12-01

**Authors:** Cynthia R. Oliveira-Martins, Cesar K. Grisolia

**Affiliations:** 1 Agência Nacional do Petróleo, Brasília, DFBrazil; 2 Departamento de Genética e Morfologia, Instituto de Ciências Biológicas, Universidade de Brasília, Brasília, DFBrazil

**Keywords:** *Allium cepa*, chromosomal aberrations, gas station, micronucleus, *Oreochromis niloticus*

## Abstract

The toxicity and genotoxicity of wastewater from eight gasoline stations in Brasília, Brazil's capital city, was studied by assessing chromosomal aberrations, chromosomal malsegregation and the mitotic index in *Alliumcepa* root cells, and the occurrence of micronucleus and nuclear abnormalities in peripheral erythrocytes of tilapia fish (*Oreochromis niloticus*). The content of gasoline station effluents was also analyzed based on several physico-chemical parameters. None of the wastewater samples was genotoxic to *A. cepa* root cells, although cell proliferation was significantly inhibited, especially at the highest concentrations. Likewise, no micronuclei were observed in *O. niloticus* peripheral erythrocytes, even after exposure to high concentrations, but there was an increase in the number of nuclear abnormalities and fish mortality. These results show that although the effluent from gasoline stations is processed by an oil/water separation system before being discharged into the main sewage system, the wastewater still contains toxic compounds.

Harmful effluents discharged into the environment have the potential to reach waterbodies and disturb aquatic ecosystems. Water contamination by gasoline residues and other petroleum derivatives is of particular concern because of the presence of polycyclic aromatic hydrocarbons (PAHs) that are mutagenic and carcinogenic (Cooke and Dennis, 1985; Hertel *etal.,* 1998). The genotoxicity of contaminated water is frequently assessed by using onion (*Allium cepa*) root cells and various fish species that provide rapid, sensitive assays for detecting genetic alterations such as micronuclei, chromosomal breaks, DNA lesion (comet assay), nuclear abnormalities and changes in the mitotic index in proliferating cells (Rank and Nielsen*,* 1993; Hayashi *et al.*, 1998; Belfiore and Anderson, 2001).

Many studies have examined the risk of genotoxicity in humans, especially gasoline station attendants, exposed to petroleum derivative compounds (Santos-Mello and Cavalcante, 1992; Carere *et al.*, 1995; Bukvic *et al.*, 1998; Çelik *et al.*, 2003; Çelik and Akbas 2005; Benites *et al.*, 2006). In contrast, there are no data on the genotoxicity of wastewater from gasoline stations. In this study, we examined the genotoxicity of wastewater from eight gasoline stations in Brasília, Brazil's capital city. We used *A. cepa* root cells to screen for chromosomal aberrations, chromosomal malsegregation and inhibition of the mitotic index, and peripheral erythrocytes of fish (*Oreochromis niloticus*; tilapia) to screen for micronuclei and nuclear abnormalities. As shown elsewhere (Grisolia and Cordeiro, 2000; Hoshima *et al.*, 2008), *O. niloticus* is a suitable species for *in situ* biomonitoring of mutagens and genotoxic compounds in effluent discharged by petroleum refineries.

As stipulated by District Acts numbers 5631 (November 27^th^, 1980) and 26590 (February 23^th^, 2006), all gasoline stations must have an effluent receptor system to separate suspended solids, oil and grease from wastewater before it goes to sewage pipelines and district wastewater treatment plants. In such a scheme, all types of petroleum residues resulting from gasoline station activities go to a system of four tanks involved in oil/water separation (Figure 1). Oil is recovered for recycling and the final effluent is discharged into the domestic sewage system. In this work, effluent samples of 20 L for physico-chemical and biological analyses were collected from the inspection tanks (Figure 1) of eight gasoline stations. The samples were analyzed according to criteria of the Brazilian Association of Technical Standards (ABNT – NBR 9800). Oil and grease were analyzed after extraction with organic solvents in a soxhlet apparatus. For each effluent sample collected, standard physico-chemical parameters were measured, including pH, biochemical oxygen demand (BOD), chemical oxygen demand (COD) and total solids in suspension. Benzene, toluene and xylene were analyzed by gas chromatography (Hewlett Packard HP 6890 gas chromatograph). Phenols, benzene, toluene and xylene were detected by spectrophotometry (Shimadzu UV420 UV-Vis spectrophotometer). Metals were detected by atomic-absorption spectrometry (Variant FS220 spectrometer).

The *A. cepa* test to screen for genotoxicity was done as described by Rank and Nielsen (1993). Commercial onion bulbs that had not been treated with growth inhibitors were obtained from organic growers. Ten onions were used for each dilution tested. Prior to the genotoxicity test, growth inhibition tests were done for each wastewater sample to determine their toxicity. Wastewater was diluted 10% and 50% and the genotoxicity tests were done for 48 h, during which the treated cell suspensions were shaken continuously in a horizontal shaker. Good quality, filtered, dechlorinated tap water (pH 7.0) was used as a negative control and for dilution of the effluents. At the end of exposure, five or six root tips from each bulb were prepared for microscopic examination. One hundred metaphase – telophase cells were analyzed per bulb (total of 1000 cells per treatment). The root tips were fixed and macerated in 45% acetic acid:1N HCl (9:1, v/v) at 50 °C for 5 min, followed by squashing in 2% orcein stain in 45% acetic acid. The slides were coded and stored in a freezer and examined within one week by an individual unaware of their identification. The root tip metaphase or anaphase cells were examined and classified as bridges, fragments, and lagging chromosomes. The mitotic index was determined by counting the number of mitotic cells (all stages) in 1000 cells.

Genotoxicity in fish was studied in aquaria containing 30 L of continuously aerated wastewater diluted 10% and 50%. The fish were maintained 24 ± 2 °C and were not fed during the exposure to wastewater. The ammonium level in the water was constantly monitored, and the conductivity and pH were kept at 500 μS and 7.2, respectively. Ten tilapias (*O. niloticus*; 30 ± 5 g, mean ± SD) from the same batch (five per aquarium) were used for each treatment during which the fish were exposed to the desired dilution for 96 h. Filtered, dechlorinated tap water was used as negative control. Peripheral blood was obtained by cardiac puncture with a heparinized syringe and immediately smeared. After fixation in ethanol for 15 min, the slides were left to air-dry and then stained with 5% Giemsa. Three thousand erythrocytes were examined for each fish at a magnification of 1000x. The nuclear abnormalities proposed by Hooftman and Raat (1982) were used as a biomarker of cytotoxicity. The nuclei were classified as blebbed, lobed, notched or binucleated. The percentage of cells with heteromorphic nuclei was determined based on an analysis of 1000 cells per fish. The frequencies of micronuclei and nuclear abnormalities were calculated from the same microscope slide.

The results were expressed as the mean ± SD. The chromosomal aberrations and changes in mitotic index in the *A. cepa* experiments were analyzed by using Students *t*-test for paired samples. The micronuclei and nuclear abnormality results with *O. niloticus* were analyzed with the non-parametric Mann Whitney–*U* test. In all cases a value of p < 0.05 indicated significance. All analyses were done using SigmaStat software, version 3.5 (Jandel Scientific).

Comparison of the physico-chemical parameters of the crude effluent in the sand tank and final effluent in the inspection tank showed that the oil/water separation system was efficient, but that it did not completely remove all of the toxic compounds in wastewater (Table 1). The levels of oil and grease were lower than reference values, indicating that the system partially removed these compounds, whereas benzene, toluene, xylene and phenols were still present in some wastewater samples. The percentage reductions for several important parameters were: COD 26%, oil and grease 6.7%, total suspended solids 50%, volatile suspended solids 41%, fixed suspended solids 59%, benzene 43%, toluene 47.7%, ethyl-benzene 62.4% and phenols 68%.

**Figure 1:**
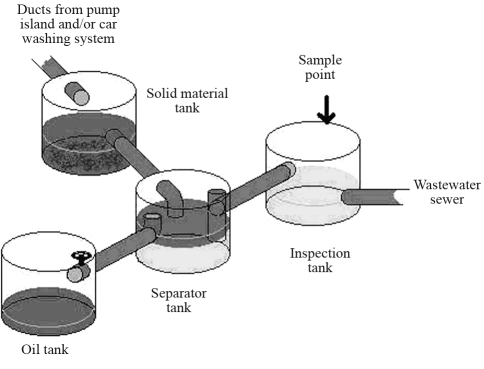
Receptor system for separating oil from water in gas station wastewater.

No chromosomal aberrations were observed with any of the samples in the genotoxicity assays with *A. cepa* (Table 2). However, cell proliferation was significantly inhibited, especially at the highest concentrations tested. There was also a significant decrease in the mitotic index of root tip cells with samples 2, 3, 5, 6, 7 and 8. Sample number 2 was the most toxic since it completely inhibited cell proliferation at a concentration of 50%. No micronuclei were observed in fish peripheral erythrocytes at any of the concentrations tested (Table 3). However, samples 2, 3, 6, 7 and 8 significantly increased the number of nuclear abnormalities in these cells (Table 4). Samples 2, 7 and 8 were tested only at a concentration of 10% because a greater concentration (50%) caused high mortality in the first 48 h of exposure.

Comparison of the values for the various parameters used to monitor the sand tanks and inspection tanks showed that although the oil/water separation system was efficient in reducing the levels of toxic compounds it did not completely remove the compounds that posed environmental risks (Table 1). The level of heavy metals, such as iron, copper, nickel and zinc, in the inspection tanks was considered insignificant. The results for the quantitative analysis of physico-chemical parameters were generally confirmed by the cyotoxicity assay in *A. cepa* root tip cells, which showed strong inhibition of cell proliferation.

The efficiency of the oil/water separation system is directly related to its regular maintenance. Based on the results obtained here, the oil/water separation systems of gasoline stations 1 and 4 showed poor maintenance because of their higher level of suspended solids. However, although these systems were saturated, their wastewater was not cytotoxic to *A. cepa* cells or genotoxic to fish erythrocytes, probably because of their low levels of benzene, toluene and xylene. In contrast, wastewater samples from gasoline stations 2, 3, 5 and 8 were the most cytotoxic to *A. cepa* because of their high content of aromatic compounds.

In conclusion, our findings indicate that although gasoline station effluents are processed before being discharged into the sewage system, the resulting wastewater still contains toxic compounds. Regular maintenance of the oil/water separation systems can help to reduce the levels of such substances in this wastewater.

## Figures and Tables

**Table 1:** Comparison of the physico-chemical parameters of samples obtained from the two main tanks of a gas station wastewater receptor system.

	Sand tank (crude effluent)	Inspection tank (final effluent)
pH	6	6
COD (mg/L)	404	299
BOD (mg/L)	180	190
Oil and grease (mg/L)	30	18
Suspended solids (mg/L)	34	17
Volatile *suspended solids* (mg/L)	17	10
Fixed *suspended solids* (mg/L)	17	7
Benzene (μg/L)	41.8	23.7
Toluene (μg/L)	291.5	152.4
Ethylbenzene (μg/L)	90.3	34.0
Xylene (μg/L)	671.4	277.1
Phenols (mg/L)	0.679	0.217
Iron (mg/L)	ND	ND
Copper (mg/L)	0.027	0.007
Nickel (mg/L)	0.105	ND
Zinc (mg/L)	0.005	0.041

ND – not detected. Solid material tank from Figure 1 correspond to sand tank.

**Table 2:** Chromosomal damage and proliferation of *A. cepa* root tip cells exposed to different concentrations of gas station wastewater.

Samples		Chromosomal aberration (%)			Total of damaged cells	p	Mitotic index (%)	p
		Bridges	Fragments	Chromosome lagging				
1	Control	0.05 ± 0.02	0.05 ± 0.03	0.00 ± 0.00	0.10 ± 0.15	-	8.83 ± 1.16	-
	10%	0.00	0.10 ± 0.32	0.00	0.10 ± 0.32	0.0732	8.89 ± 1.94	0.8203
	50%	0.00	0.15 ± 0.67	0.10 ± 0.32	0.25 ± 0.40	0.4345	7.56 ± 1.70	0.1038

2	Control	0.00	0.00	0.00	0.00	-	10.75 ± 2.47	-
	10%	0.12 ± 0.67	0.05 ± 0.48	0.10 ± 0.32	0.27 ± 0.87	0.0303	3.12 ± 1.51	0.0041*

3	Control	0.00	0.00	0.10 ± 0.32	0.10 ± 0.32	-	9.47 ± 2.89	-
	10%	0.00	0.00	0.10 ± 0.32	0.10 ± 0.32	0.5032	7.80 ± 1.77	0.1734
	50%	0.00	0.00	0.14 ± 0.12	0.14 ± 0.12	0.5270	2.58 ± 0.79	0.0046*

4	Control	0.10 ± 0.32	0.05 ± 0.12	0.00	0.15 ± 0.50	-	9.80 ± 2.96	-
	10%	0.10 ± 0.32	0.10 ± 0.32	0.10 ± 0.32	0.30 ± 0.67	1.0000	11.27 ± 2.14	0.1433
	50%	0.06 ± 0.42	0.12 ± 0.42	0.02 ± 0.20	0.20 ± 0.71	0.3986	11.07 ± 1.52	0.1647

5	Control	0.00	0.00	0.00	0.00	-	10.43 ± 3.92	-
	10%	0.00	0.18 ± 0.23	0.00	0.18 ± 0.23	0.1256	4.23 ± 2.80	0.0048*
	50%	0.00	0.00	0.00	0.00	0.0000	1.04 ± 0.49	0.0006*

6	Control	0.00	0.10 ± 0.32	0.02 ± 0.42	0.12 ± 0.35	-	10.43 ± 3.32	-
	10%	0.00	0.00	0.10 ± 0.32	0.10 ± 0.32	0.2758	10.57 ± 3.69	0.7623
	50%	0.08 ± 0.30	0.18 ± 0.13	0.00 ± 0.00	0.26 ± 0.22	0.8420	4.01 ± 1.60	0.0112*

7	Control	0.00	0.00	0.00	0.00	-	14.08 ± 1.40	-
	10%	0.11 ± 0.33	0.11 ± 0.33	0.00	0.22 ± 0.67	0.0820	4.31 ± 2.12	0.0128*
	50%	0.14 ± 0.60	0.10 ± 0.52	0.17 ± 0.41	0.41 ± 0.97	0.0538	5.12 ± 1.84	0.0199*

8	Control	0.02 ± 0.42	0.05 ± 0.63	0.10 ± 0.95	0.17 ± 0.87	-	12.32 ± 3.44	-
	10%	0.00	0.10 ± 0.32	0.10 ± 0.32	0.20 ± 0.42	0.4853	9.65 ± 3.74	0.6968
	50%	0.00	0.00	0.14 ± 0.38	0.14 ± 0.38	0.3930	2.57 ± 0.66	0.0065*

*Significantly different (p < 0.05) from the control.

**Table 3:** Occurrence of micronuclei and nuclear abnormalities in peripheral erythrocytes of *O. niloticus* exposed to gas station wastewater.

Samples	Dilution (%)	Micronuclei per 3000 cells	p	Nuclear abnormalities per 1000 cells	p
Control	-	1.10 ± 1.37		69.2 ± 32.7	
1	10	6.82 ± 4.69	0.3012	68.6 ± 31.8	0.8879
	50	2.00 ± 1.90	0.1903	88.6 ± 45.4	0.2311

2	10	0.45 ± 0.82	0.6500	188.6 ± 13.8	0.0211*

3	10	0.58 ± 0.67	0.5653	115.3 ± 41.1	0.0372*
	50	0.20 ± 0.42	0.9000	174.9 ± 23.0	0.0163*

4	10	3.70 ± 2.63	0.1045	89.6 ± 42.7	0.2566
	50	3.40 ± 1.90	0.1090	60.1 ± 19.7	0.4725

5	10	3.90 ± 1.73	0.0924	50.8 ± 17.1	0.3471
	50	2.20 ± 1.55	0.0748	51.9 ± 15.7	0.2315

6	10	1.45 ± 1.21	0.3588	86.9 ± 27.9	0.0649
	50	1.78 ± 1.72	0.3920	163.8 ± 27.9	0.0149*

7	10	2.67 ± 1.50	0.2158	227.7 ± 53.6	0.0011*

8	10	3.70 ± 2.58	0.0354	170.9 ± 30.7	0.0297*

*Significantly different (p < 0.05) from the control.

## References

[BelfioreandAnderson2001] Belfiore N.M., Anderson S.L. (2001). Effects of contaminants on genetic patterns in aquatic organisms: A review. Mutat Res.

[Benitesetal2006] Benites C.I., Amado L.L., Vianna R.A.P., Martino-Roth M.G. (2006). Micronucleus test on gas attendants. Genet Mol Res.

[Bukvicetal1998] Bukvic N., Bavaro P., Elia G., Cassano F., Fanelli M., Guanti G. (1998). Sister chromatid exchanges (SCE) and micronucleus (MN) frequencies in lymphocytes of gasoline station attendants. Mutat Res.

[Carereetal1995] Carere A., Antoccia A., Crebelli R., Degrassi F., Fiore M., Iavarone I., Isacchi G., Lagorio S., Leopardi P., Marcon F. (1995). Genetic effects of petroleum fuels: Cytogenetic monitoring of gasoline station attendants. Mutat Res.

[CelikandAkbas2005] Çelik A., Akbas E. (2005). Evaluation of sister chromatid exchanges and chromosomal aberration frequencies in peripheral blood lymphocytes of gasoline station attendants. Ecotox Environ Saf.

[Celiketal2003] Çelik A., Çavas T., Ergene-Gozukara S. (2003). Cytogenetic biomonitoring in petrol station attendants: Micronucleus test in exfoliated buccal cells. Mutagenesis.

[EnvironmentalProtectionAgency1985] Cooke M., Dennis A.J., Environmental Protection Agency (1985). Polynuclear aromatic hydrocarbons: Mechanism, method and metabolism. American Petroleum Institute.

[GrisoliaandCordeiro2000] Grisolia C.K., Cordeiro C.M.T. (2000). Variability in micronucleus induction with different mutagens applied to several species of fish. Genet Mol Biol.

[Hayashietal1998] Hayashi M., Ueda T., Uyeno K., Wada K., Kiane N., Saotorne K., Tanaka N., Takai A., Sasaki Y.F., Asano N. (1998). Development of genotoxicity assay systems that use aquatic organisms. Mutat Res.

[InternationalProgramonChemicalSafety1998] Hertel R.F., Rosner G., Kielhorn J., International Program on Chemical Safety (1998). Selected non-heterocyclic aromatic hydrocarbons. , Environmental Health Criteria 202.

[HooftmanandRaat1982] Hooftman R.N., Raat W.K. (1982). Induction of nuclear abnormalities (micronuclei) in the peripheral blood erythrocytes of the eastern mudminnow *Umbra pygmaea* by ethyl methanesulphonate. Mutat Res.

[Hoshimaetal2008] Hoshima M.M., Angelis D.F., Marin-Morales M.A. (2008). Induction of micronucleus and nuclear alterations in fish (*Oreochromis niloticus*) by a petroleum refinery effluent. Mutat Res.

[RankandNielsen1993] Rank J., Nielsen M.H. (1993). A modified *Allium* test as a tool in the screening of genotoxicity of complex mixtures. Hereditas.

[Santos-MelloandCavalcante1992] Santos-Mello R., Cavalcante B. (1992). Cytogenetic studies on gas station attendants. Mutat Res.

